# How factors connected to the natural environment shape football fans’ eudaimonic and hedonic well-being

**DOI:** 10.3389/fpsyg.2025.1604029

**Published:** 2025-09-30

**Authors:** Christian Kraft, Christoph Bühren, Pamela Wicker

**Affiliations:** ^1^Department of Sports Science, Bielefeld University, Bielefeld, Germany; ^2^Faculty of Sport Science, Ruhr University Bochum, Bochum, Germany

**Keywords:** connectedness to nature, perceived environmental pollution, transport-specific environmental consciousness, environmental knowledge, psychological well-being, football fans

## Abstract

**Introduction:**

Football fans contribute to pollution and environmental impact, yet how they perceive factors connected to the natural environment remains understudied. This study focuses on four such factors, including connectedness to nature, perceived environmental pollution, transport-specific environmental consciousness, and environmental knowledge, that are particularly relevant in the context of sports-related behavior. Understanding these perceptions is important, especially as environmental education alone often falls short in fostering sustained pro-environmental behavior. This connection is crucial, as individuals may be more inclined to adopt environmentally sustainable behaviors when such actions are perceived to support, or at least not undermine, their well-being. This study examines how factors connected to the natural environment relate to eudaimonic and hedonic well-being among football fans in Germany.

**Methods:**

Survey data was collected from 839 football fans of a German third-division club. Well-being was assessed using validated scales for eudaimonic and hedonic well-being. Seemingly unrelated regression models were employed to examine how factors connected to the natural environment relate to the two well-being measures, controlling for demographic variables.

**Results:**

The findings indicated that, among fans, the assessed factors connected to the natural environment were moderately pronounced. Connectedness to nature and environmental knowledge scored slightly higher, while perceived environmental pollution and transport-specific environmental consciousness showed neutral ratings. Connectedness to nature was positively associated with both eudaimonic and hedonic well-being. Perceived environmental pollution was negatively linked to hedonic well-being, and transport-specific environmental consciousness was negatively related to eudaimonic well-being. No significant associations were found for environmental knowledge.

**Discussion:**

These results highlight the complex role of environmental perceptions in shaping football fans’ well-being. While fostering connectedness to nature may enhance overall well-being, sustainability concerns may lead to psychological burdens. Investigating these dynamics in a population often viewed as environmentally indifferent challenges common stereotypes and reveals that football fans are aware of environmental issues. By understanding these perceptions, stakeholders can design sustainability initiatives that build on fans’ existing values and behaviors, encouraging meaningful participation and environmentally responsible matchday practices that also support well-being.

## 1 Introduction

Football fans are often perceived – at least on match days – as disruptive elements in urban environments (Pearson, 2012). Many city residents associate them with rowdy behavior, public intoxication, littering, and vandalism, as well as the need for increased police presence to manage large crowds (Stott and Pearson, 2007; Stott, 2014). In Germany alone, policing football-related operations during the 2023/24 Football Bundesliga season required the equivalent of 1,572 full-time police officers, illustrating the substantial resource allocation and financial burden associated with match-day security (Zentralstelle für Sporteinsätze, 2024). Furthermore, stadium-related waste contributes significantly to urban pollution, with an estimated 750,000 tons of waste generated annually by major football events in Europe (Union of European Football Associations, 2023). From this perspective, football fans represent a temporary environmental burden on society coupled with economic costs, requiring substantial policing, transportation logistics, and environmental cleanup efforts (Armstrong and Giulianotti, 2002). These observed behaviors, along with public perception, raise the question of whether observed behaviors genuinely reflect football fans’ underlying environmental attitudes or whether social dynamics specific to match days temporarily influence their behavior without necessarily representing their personal environmental values.

Generally speaking, sporting events have substantial environmental impacts, primarily due to fan travel, waste generation, and extensive resource consumption (Cerezo-Esteve et al., 2022). Sports organizations, including football clubs, have begun to implement environmental sustainability initiatives aimed at reducing emissions, promoting eco-friendly transportation, and encouraging pro-environmental attitudes among their fans (Cayolla et al., 2021; Kraft et al., 2024; McCullough et al., 2015; Trendafilova et al., 2013). These efforts are often framed as necessary steps to mitigate climate change, but they may also have psychological and behavioral implications for fans. Specifically, sustainability initiatives may raise fans’ awareness of environmental issues, such as the impact of matchday travel, through which fans develop a stronger environmental consciousness and more pronounced attitudes (Kraft et al., 2024; McCullough and Cunningham, 2010).

However, the effectiveness of such initiatives and their broader impact on fan well-being remain largely unexplored. Football fans are known to contribute to pollution and environmental impact, yet little is known about how they perceive and engage with factors connected to the natural environment, and how these relate to their well-being. In the present study, we define these factors as individual-level psychological constructs, including perceptions, knowledge, beliefs, and attitudes, related to the natural environment. Specifically, we examine connectedness to nature, perceived environmental pollution, transport-specific environmental consciousness, and environmental knowledge. For conciseness, we refer to them collectively as factors connected to the natural environment throughout the study. While sustainable practices may foster a sense of alignment with personal values and collective responsibility, they may also introduce tensions, such as eco-anxiety or cognitive dissonance (Kurth and Pihkala, 2022). Rather than examining situational behaviors on match days, this study focuses on how football fans generally perceive environmental issues and how these perceptions shape their well-being. Only by understanding these connections can environmental concerns become personally relevant and potentially influence pro-environmental attitudes and behaviors, also on match days.

Given that environmental perceptions, such as connectedness to nature, pollution concerns, or climate awareness, are closely linked to psychological states (Kals et al., 1999; Thomson and Roach, 2023), it is essential to examine their direct associations with the well-being of football fans. Despite growing attention to fan well-being, its conceptualization and measurement remain inconsistent (Trainor and Bundon, 2023), leaving critical gaps in understanding how it relates to factors connected to the natural environment. Well-being involves a complex interplay of factors, encompassing both eudaimonic well-being (e.g., finding personal meaning, purpose, and engaging deeply with one’s values) and hedonic well-being (e.g., experiencing short-term enjoyment and positive emotions) (Baumeister et al., 2013). While eudaimonic well-being is associated with long-term personal growth and alignment with values, hedonic well-being focuses on immediate happiness and alleviation of negative emotions (Ryan and Deci, 2001). Fan well-being is shaped by various factors, including social identity, community belonging, and external conditions (Kesler and Wann, 2020). Among these, factors connected to the natural environment – such as connectedness to nature, perceived environmental pollution, transport-specific environmental consciousness, and environmental knowledge – may play a distinct role in influencing how fans experience well-being. Fans with high environmental consciousness may experience cognitive dissonance when confronted with the ecological impact of football culture, regardless of their general affinity for the sport (Langseth and Vyff, 2021).

The purpose of this study is to investigate how specific factors connected to the natural environment relate to football fans’ eudaimonic and hedonic well-being. Specifically, this study examines four key factors of them: connectedness to nature – the extent to which individuals feel emotionally attached to nature, potentially enhancing well-being through relaxation and alignment with personal values (Ryan and Deci, 2001); perceived environmental pollution – the subjective perception of pollution (e.g., air quality, noise, waste), potentially acting as a psychological stressor (Preisendörfer, 1999); transport-specific environmental consciousness – the degree of awareness regarding the environmental impacts of personal travel behavior, influencing well-being through trade-offs between convenience and sustainability (Loewen and Wicker, 2021; Preisendörfer, 1999), and environmental knowledge – familiarity with environmental problems and action strategies, possibly reducing climate anxiety but also heightening awareness and distress (Geiger et al., 2019; Zacher and Rudolph, 2023). The study addresses the following research question (RQ): How do factors connected to the natural environment – connectedness to nature, perceived environmental pollution, transport-specific environmental consciousness, and environmental knowledge – relate to the eudaimonic and hedonic well-being of football fans? Although happiness and a meaningful life overlap, this study focuses on the potentially different antecedents driving these two dimensions of well-being.

The research question is answered using data from an online survey with fans of a German third-division Football club. The league averaged 10,000 spectators per game in the 2023/2024 season (Deutscher Fußball Bund, 2024). The research aims to uncover generalizable insights into how factors connected to the natural environment relate to fan well-being, particularly in contexts where sustainability issues are increasingly prominent. The findings offer practical guidance to football club managers, sponsors, and policymakers on how sustainability initiatives can shape these factors in ways that promote fan well-being. By addressing aspects such as connectedness to nature, perceived environmental pollution, transport-specific environmental knowledge, and environmental knowledge, such initiatives may be more effective if they align with the factors that enhance well-being. This study integrates insights from sports research, environmental psychology, and sustainability studies, providing a nuanced understanding of how football fans engage with the natural environment and how this relates to their well-being, challenging assumptions about their environmental indifference and highlighting opportunities for more impactful sustainability strategies in sport.

## 2 Theoretical background and literature review

Well-being is broadly defined as a person’s overall quality of life and psychological functioning (Diener et al., 1985). It encompasses both subjective experiences of happiness and deeper feelings of meaning and purpose in life. Within the well-being research, two key dimensions have been identified: hedonic well-being, which focuses on pleasure and positive emotions, and eudaimonic well-being, which relates to living in accordance with one’s values and personal growth (Ryan and Deci, 2001). Eudaimonic well-being and hedonic well-being are distinct but strongly correlated (Huta and Ryan, 2010; Ryan and Deci, 2001). Eudaimonic (or hedonic) activities likely influence and nurture the development of hedonic (or eudaimonic) well-being (Fredrickson, 2004). Both perspectives of well-being originate from philosophical deliberations.

### 2.1 Eudaimonic well-being

Eudaimonic well-being originates in the philosophical assumptions of Aristotle about eudaimonia. Aristotle (1925) described eudaimonia as a result of living in agreement with one’s true nature. Individuals with high levels of eudaimonic well-being live according to their values and realize their full potential, which makes their lives more meaningful (Waterman, 2008). Eudaimonic well-being is conceptualized as a multidimensional construct (Deci and Ryan, 2008; Huta, 2015; Ryff, 1989). It encompasses several key components (Deci and Ryan, 2008; Huta, 2015; Ryff, 1989):

1. Personal growth reflects the continual development of one’s potential.
2. Purpose in life is characterized by having goals, direction, and a sense of meaning.
3. Feelings of meaningfulness represent the belief that life has value and significance.
4. Autonomy involves self-determination and independence in regulating one’s behavior.
5. Environmental mastery refers to the ability to manage and shape one’s environment to meet personal needs.
6. Self-acceptance entails holding positive attitudes toward oneself and one’s past.
7. Positive relations with others emphasize warm, trusting, and meaningful interpersonal connections.

Activities related to eudaimonic well-being are meaningful and offer long-term benefits, encompassing reflections on the past, present, and future (Baumeister et al., 2013; Huta and Ryan, 2010; Ryan and Deci, 2001). Moreover, individuals’ eudaimonic well-being benefits from a balance between a self-focus and a focus on and contributing to others, society, and the natural environment. Eudaimonic activities and lifestyles can be challenging and less pleasurable at times, however, they provide personal meaning (Baumeister et al., 2013).

### 2.2 Hedonic well-being

The philosophical origins of hedonic well-being trace back to Aristippus, who described hedonia as maximizing pleasure and avoiding pain (Ryan and Deci, 2001). Hedonic well-being refers to the subjective experience of happiness, pleasure, and positive emotions while minimizing negative emotions (Huta and Ryan, 2010; Ryff, 1989). It is centered on satisfaction, enjoyment, and the pursuit of pleasure. Hedonic well-being consists of three primary components: positive affect, negative affect, and life satisfaction (Deci and Ryan, 2008; Ryff et al., 2021). Positive affect refers to the emotions and moods individuals experience, such as joy and excitement, when things are going well. It reflects a component of hedonic well-being, as it represents an individual’s evaluation that life is proceeding favorably (Diener et al., 2017; Ryan and Deci, 2001). On the other hand, negative affect refers to emotions and moods, such as anger and anxiety, that arise when individuals have negative experiences (Huta and Ryan, 2010; Trainor and Bundon, 2023). It is a component of hedonic well-being because it reflects an individual’s negative evaluation of life and health. Life satisfaction is a cognitive evaluation of one’s overall quality of life, based on personal standards and expectations. It reflects how satisfied individuals are with their lives and represents the cognitive component of hedonic well-being (Deci and Ryan, 2008; Ryan and Deci, 2001). From a hedonic perspective, individuals prioritize maximizing pleasure and minimizing pain, often focusing on themselves and the present moment rather than helping others or considering long-term impacts (Huta and Ryan, 2010; Huta, 2015).

### 2.3 Factors affecting well-being

A wide range of constructs has been proposed to capture individual-level factors connected to the natural environment that are relevant to behavior and well-being, including values, attitudes, identity, and knowledge (Kollmuss and Agyeman, 2002). In this study, we focus on the four presented determinants. These were chosen because they reflect key affective aspects (e.g., feeling connected to nature), cognitive dimensions (e.g., knowledge and awareness of environmental problems), and behavioral components (e.g., willingness to engage in sustainable mobility) which are commonly identified as core drivers of environmental behavior and different well-being dimensions (Kollmuss and Agyeman, 2002; Li et al., 2018; Pritchard et al., 2020). At the same time, these constructs are especially relevant for the context of sports fandom and mobility, where environmental attitudes may be closely linked to travel behavior and place-based experiences. While many other determinants could have been included, our aim was to cover different but complementary perspectives on factors connected to the natural environment without making the model overly complex.

Although the four determinants reflect distinct factors connected to the natural environment, they are conceptually interrelated and may operate as a broader system of environmentally oriented attitudes and cognitions. Connectedness to nature, for example, is often regarded as an emotional foundation that can foster greater concern for environmental issues and stimulate interest in environmental knowledge (Otto and Pensini, 2017). Although environmental knowledge is sometimes assumed to emerge from such connectedness, studies have shown that the two constructs are only weakly correlated (Otto and Pensini, 2017; Roczen et al., 2013). Early research suggested that environmental knowledge is not a core component of environmental consciousness (Maloney and Ward, 1973), but rather a distinct construct (Preisendörfer, 1999). Later frameworks, however, acknowledge its partial independence while still recognizing its role in shaping environmental awareness (Kollmuss and Agyeman, 2002). Grob (1991), for instance, argues that knowledge can contribute to awareness. Similarly, Chawla (1999) emphasizes that emotional connection to nature, more than knowledge alone, often drives the development of environmental consciousness. Taken together, these perspectives suggest that while the determinants are conceptually linked, they reflect distinct yet complementary factors connected to the natural environment.

The two dimensions of well-being may be linked to different predictors (Baumeister et al., 2013) due to their distinct theoretical foundations. Previous research has extensively examined determinants such as income, social relationships, personality traits, and physical health in relation to both eudaimonic and hedonic well-being (Deci and Ryan, 2008; Diener et al., 2017; Huta and Ryan, 2010; Huta, 2015; Ryff et al., 2021; Ryff, 1989; Waterman, 2008). However, some predictors may be more relevant for one dimension than for the other, reflecting the conceptual differences between long-term meaning-oriented and short-term pleasure-oriented well-being. Additionally, some potential determinants have remained largely overlooked. These include connectedness to nature, perceived environmental pollution, transport-specific environmental consciousness, and environmental knowledge.

Connectedness to nature reflects the extent to which individuals have an affective relationship with the natural world (Kals et al., 1999; Kollmuss and Agyeman, 2002; Whitburn et al., 2020). In other words, the concept encompasses subjective evaluations of individuals’ emotional bond with nature (Whitburn et al., 2020). The Biophilia Hypothesis (Wilson, 1984) suggests that humans possess an innate biological attraction to nature, rooted in evolutionary history. This connection is essential for well-being because humans evolve in natural environments that provide safety, resources, and survival advantages (Kollmuss and Agyeman, 2002; Nisbet et al., 2009). Modern environments, where nature is diminished, can disrupt this connection and be detrimental to both physical and mental health (Beery et al., 2023; Nisbet et al., 2009). Individuals with a stronger connectedness to nature are often argued to have higher well-being because their lifestyles and environments may align more closely with human evolutionary needs, which remain embedded in our psychology (Mayer and Frantz, 2004; Wilson, 1984). In addition to the Biophilia Hypothesis, the stress reduction theory (Ulrich et al., 1991) explains this relationship by highlighting the immediate physiological and emotional benefits of nature exposure. According to stress reduction theory, viewing or spending time in natural environments triggers the parasympathetic nervous system, leading to reduced stress, lower cortisol levels, and improved mood (Ulrich et al., 1991). Natural settings evoke positive emotional responses, such as calmness and relaxation, which enhance overall well-being.

The Biophilia Hypothesis and the stress reduction theory emphasize that the connection between nature and human well-being is not only psychological, but also biological. The disconnection from nature can harm physical and mental health while fostering behaviors and attitudes that contribute to environmental degradation (Mayer and Frantz, 2004; Nisbet et al., 2009). Thus, nurturing a strong relationship with nature is vital for both human well-being and environmental sustainability. Previous research has consistently shown a positive relationship between connectedness to nature and various well-being measures (Capaldi et al., 2014; Howell et al., 2011; Nisbet and Zelenski, 2013; Pritchard et al., 2020). Meta-analyzes have demonstrated links to both hedonic and eudaimonic well-being in general populations (Capaldi et al., 2014; Pritchard et al., 2020). Other studies have explored associations with mindfulness and personality traits (Howell et al., 2011) or focused on the development of measurement tools for connectedness to nature (Nisbet and Zelenski, 2013). However, these investigations have been conducted outside the context of sports and football fandom. The extent to which connectedness to nature influences well-being specifically among football fans remains an open question and warrants investigation.

The strength of these effects may depend on the dimension of well-being (eudaimonic vs. hedonic). Connectedness to nature could relate more strongly to eudaimonic well-being: It encompasses meaningfulness, a sense of being part of something greater, and personal involvement, which are key aspects of eudaimonic well-being (Pritchard et al., 2020). Additionally, higher connectedness to nature often develops through frequent nature experiences. In light of the Biophilia Hypothesis, this could reflect living in alignment with one’s true nature, a core element of eudaimonic well-being. Moreover, eudaimonic well-being is generally associated with long-term behavioral patterns, which also align with connectedness to nature, as it is known to increase over time and with repeated exposure to nature.

Previous well-being research partially supports these assumptions: For example, Howell et al. (2011) found that connectedness to nature was more strongly correlated with eudaimonic well-being than with hedonic well-being. Capaldi et al. (2014) reported higher effect sizes for vitality – a measure of eudaimonic well-being – compared to positive affect and life satisfaction, which are more closely related to hedonic well-being. However, a meta-analysis by Pritchard et al. (2020) found no significant differences in effect sizes between eudaimonic and hedonic well-being studies, raising questions about the consistency of these differences. Hypothesis 1a aligns with theoretical tenets, suggesting that individuals with greater connectedness to nature experience higher well-being. While some empirical findings are contradictory, Hypothesis 1b posits that the relationship between connectedness to nature and eudaimonic well-being is stronger than its association with hedonic well-being:

H1a: Connectedness to nature is positively associated with eudaimonic and hedonic well-being.

H1b: Connectedness to nature is more strongly associated with eudaimonic well-being than with hedonic well-being.

Perceived environmental pollution refers to individuals’ subjective evaluations and awareness of pollution within their surroundings, which is shaped by personal observations, experiences, and sensitivities (Preisendörfer, 1999; Yang, 2020). These perceptions can be influenced by various types of pollution, including air pollution, noise pollution, light pollution, and visible litter. Factors such as visibility, odors, and noise contribute to these assessments, which may not always correspond with objective measurements.

Research suggests that stress results from the interaction of psychological and biological factors, which may explain the link between perceived environmental pollution and well-being (Bullinger, 1989; Lazarus and Cohen, 1977). Stress arises in response to external or internal stressors that disrupt an individual’s equilibrium, involving both physiological processes (e.g., hormonal changes, increased heart rate) and psychological responses (e.g., anxiety or perceived threat) (Lazarus and Cohen, 1977). Although environmental pollution is generally seen as a threat to quality of life, especially in terms of comfort or health, its impact on eudaimonic well-being may be more complex and deserves closer examination (Gu et al., 2015).

According to the meaning maintenance model (Heine et al., 2006), negative environmental conditions such as air pollution can challenge an individual’s fundamental belief in a stable and safe environment, thereby violating their sense of meaning (Proulx et al., 2013). In response to such disruptions, individuals may attempt to reaffirm meaning in other life domains, a process known as compensatory affirmation (Van Tongeren and Green, 2010). This could manifest in stronger engagement with goals related to personal growth or life purpose, central aspects of eudaimonic well-being. Empirical support for this idea comes from a study among Beijing residents, which found positive associations between perceived air pollution and eudaimonic well-being (Gu et al., 2015). This suggests that confronting environmental challenges may sometimes catalyze deeper reflection or goal pursuit. However, these findings remain counterintuitive and context-dependent, and the scope of existing evidence is limited, often focused on air pollution alone, without considering other stressors like noise or litter, and with a focus on hedonic well-being (Álvarez et al., 2023). Given the scarcity of research on eudaimonic outcomes, related constructs such as ill-being (e.g., depression, anxiety) may offer indirect insights (Keyes, 2005). Several studies have found that air pollution is associated with increased depressive symptoms and psychological distress (Braithwaite et al., 2019; Costa et al., 2020; Newbury et al., 2019). However, a large cohort study (Zijlema et al., 2016) failed to detect consistent associations between air pollution and ill-being measures, leading to discussions about the strength and consistency of this relationship (Kawada, 2016). These mixed results underscore the need for more research into how environmental stressors may, in some cases, evoke adaptive or meaning-driven responses that support eudaimonic well-being.

The effects of perceived environmental pollution on hedonic well-being can be viewed from a different perspective. Perceived pollution can function as a psychological stressor, as individuals may interpret polluted environments (e.g., air pollution) as a threat to their health (Bullinger, 1989; Campbell, 1983). Notably, this perception can induce stress even when objective pollution levels are not immediately harmful, as mere awareness or belief in pollution can trigger psychological discomfort. Furthermore, the continuous perception of living in a polluted environment can elicit negative emotions, such as sadness, anger, or hopelessness, which can substantially diminish hedonic well-being (Li et al., 2018). Research has shown that perceived environmental pollution negatively affects hedonic well-being. So far, most research has focused on air quality as a measure of pollution (Gu et al., 2015; Herrera and Cabrera-Barona, 2020; Li et al., 2018; Rehdanz and Maddison, 2008). Relationships with other dimensions of pollution have received less attention. However, existing research indicates that noise pollution is associated with reduced hedonic well-being (Hammersen et al., 2016; Herrera and Cabrera-Barona, 2020). Similar negative effects have been found for exposure to unpleasant smells (Bentley et al., 2023; Finell et al., 2024) and visible litter (Hassan and Khalil, 2024). Additionally, studies using a composite measure of perceived environmental pollution suggest that overall pollution perception negatively correlates with hedonic well-being (Li and Zhou, 2020). All these studies include measures of hedonic well-being (i.e., life satisfaction, positive affect, negative affect), neglecting the eudaimonic perspective of well-being.

While perceived environmental pollution may inspire individuals to confront environmental challenges in ways that promote eudaimonic well-being, it can also undermine hedonic well-being by inducing stress and negative emotions (Campbell, 1983; Gu et al., 2015). In some cases, the perception of environmental problems may prompt individuals to engage in activism, community initiatives, or sustainable practices (Eusébio et al., 2023). These value-driven responses can foster a sense of purpose or accomplishment, which may support eudaimonic well-being (Heine et al., 2006; Proulx et al., 2013; Van Tongeren and Green, 2010). Balancing this view, Xu et al. (2017) found that environmental risk perception, defined as the awareness and anticipation of environmental threats, negatively influenced both hedonic and eudaimonic well-being in a Chinese sample. Although risk perception and perceived environmental pollution are conceptually related, as both involve subjective evaluations of environmental threats, they differ in emphasis. Risk perception typically includes broader, anticipatory judgments about severity and characteristics of risks (Xu et al., 2017), whereas perceived environmental pollution refers to awareness of environmental pollution such as visible litter or unpleasant smells (Yang, 2020). The relationship between perceived environmental pollution and well-being is particularly relevant in the context of sports fans, where pollution perceptions may interact with identity or place attachment (McCullough and Kellison, 2016). At the same time, the continuous perception of living in a polluted environment can elicit negative emotions, such as sadness, anger, or hopelessness, which may substantially diminish hedonic well-being (Li et al., 2018). These aspects are addressed by the second hypothesis. Given the mixed previous findings and conceptual nuances, the relationship between perceived environmental pollution and eudaimonic well-being remains tentative:

H2a: Perceived environmental pollution is positively associated with eudaimonic well-being.

H2b: Perceived environmental pollution is negatively associated with hedonic well-being.

Previous research has also identified a link between environmental consciousness and well-being (Thormann et al., 2022). Environmental consciousness refers to an individual’s awareness and comprehension of environmental challenges, their understanding of the consequences of human actions on ecosystems, and their emotional engagement with environmental issues (Preisendörfer, 1999). In the present study, environmental consciousness is conceptualized using a three-dimensional model (Diekmann and Preisendörfer, 2003; Preisendörfer, 1999; Thormann et al., 2022), consisting of the affective dimension, which reflects emotional responses such as concern or distress about environmental problems; the cognitive dimension, which encompasses knowledge of environmental threats and the risks associated with unsustainable behavior; and the conative dimension, which represents the motivation and willingness to engage in environmentally responsible actions. While this framework has been adopted in prior studies (Diekmann and Preisendörfer, 2003; Preisendörfer, 1999; Thormann et al., 2022), it represents one of several possible approaches to measuring environmental consciousness.

In the context of football fandom, transport-specific environmental consciousness represents a particularly relevant dimension of environmental consciousness. Given that fan travel constitutes one of the most significant environmental impacts of football events, transport-related environmental consciousness may be a crucial factor for fans, which might also shape their well-being. Unlike general environmental consciousness, which broadly includes awareness, attitudes, and concerns about environmental issues, transport-specific environmental consciousness focuses specifically on individuals’ awareness of the ecological consequences of their travel choices, their willingness to adopt more sustainable transport behaviors, and their emotional responses to mobility-related environmental issues. Investigating this construct allows for a more targeted understanding of how environmental concerns affect well-being in the context of travel and transport.

Building on the work of Ferrer-I-Carbonell and Gowdy (2007), environmental consciousness has been found to influence well-being in both positive and negative ways. The direction of this effect largely depends on how individuals perceive and interpret their environmental consciousness. On the one hand, positive associations, such as engaging in biodiversity conservation, can contribute to higher well-being. On the other hand, negative associations, such as distress over pollution and environmental destruction, may lead to lower well-being (Binder and Blankenberg, 2016; Suárez-Varela et al., 2016). Similar patterns may apply to transport-specific environmental consciousness. While adopting sustainable travel behaviors might foster a sense of alignment with personal values and enhance eudaimonic well-being, the awareness of transport-related emissions and personal mobility choices could also induce guilt or frustration, potentially reducing hedonic well-being.

Empirical studies on this relationship have yielded mixed results across different contexts. For instance, Rehdanz and Maddison (2008) analyzed environmental quality in Germany and found that concerns about local pollution negatively affected life satisfaction. Similarly, Thormann et al. (2022) investigated football fans’ stadium travel behavior and reported a negative impact of environmental consciousness on life satisfaction and happiness, highlighting the emotional burden of environmental awareness in a sports-related setting. Conversely, studies by Binder and Blankenberg (2016), who examined environmental activism in Germany, and Nisbet et al. (2011), who explored nature relatedness, identified a positive relationship between environmental consciousness and well-being. With a Spanish sample, Suárez-Varela et al. (2016) also found that pro-environmental behaviors contributed to well-being. Further evidence suggested that both positive and negative effects can coexist, depending on how individuals emotionally and cognitively frame environmental issues (Ferrer-I-Carbonell and Gowdy, 2007).

Although these findings confirm a connection between environmental consciousness and well-being, the direction and strength of the relationship remain uncertain. A key factor in these inconsistencies may be the type of well-being measure and the corresponding perspective of well-being – whether studies focus on eudaimonic well-being (e.g., meaning and personal growth) or hedonic well-being (e.g., life satisfaction and positive emotions). Another potential reason for inconsistent findings may lie in the operationalization of environmental consciousness itself. Most previous research has used general measures of environmental consciousness, whereas research on more specific forms, such as transport-specific environmental consciousness, is still lacking. This represents a relevant research gap, particularly in the context of football fans, as travel behavior constitutes one of the most significant environmental impacts of football events. Accordingly, transport-related environmental consciousness may be especially relevant in this context and could shape well-being through perceived dissonance between values and behavior. Transport-specific environmental consciousness could be positively associated with eudaimonic well-being. While the conative dimension, reflecting the willingness to adopt more sustainable transport behaviors, might align with key components of eudaimonic well-being, such as purpose or acting according to personal values, such benefits may only materialize when individuals are able to act on these intentions. In settings where fans are unable to adopt sustainable alternatives, this alignment may be weakened or disrupted. However, previous research has shown that value alignment and moral self-identity are associated with personal growth and meaning, even when behavioral enactment is limited (Schwartz et al., 2012; Sheldon and Kasser, 2001). Thus, holding strong transport-related environmental consciousness may foster eudaimonic well-being through internal coherence and perceived integrity. Additionally, aspirational thinking and goal commitment have been linked to higher eudaimonic well-being, as they promote a future-oriented mindset and a sense of agency (Baumeister et al., 2013; McKnight and Kashdan, 2009). In this light, the awareness of environmental impacts from mobility and the motivation to adopt more sustainable transport behaviors, core elements of transport-specific environmental consciousness, may support feelings of purpose and personal growth, even if individuals are not immediately able to act on these intentions. Conversely, for hedonic well-being, the affective and cognitive dimensions of transport-specific environmental consciousness may trigger negative emotional responses, such as guilt or frustration, especially when fans are aware of the environmental impact of their travel behavior but feel unable to change it. This dissonance may diminish hedonic well-being by inducing feelings of helplessness or eco-anxiety (Schwartz et al., 2022; Searle and Gow, 2010). This leads to the following hypotheses:

H3a: Transport-specific environmental consciousness is positively associated with eudaimonic well-being.

H3b: Transport-specific environmental consciousness is negatively associated with hedonic well-being.

Finally, environmental knowledge refers to an individual’s understanding of environmental issues, their underlying causes, and possible solutions (Fryxell and Lo, 2003; Geiger et al., 2019). It includes factual knowledge about ecosystem processes, functions, and structures, and it is typically categorized into four key dimensions: awareness of environmental issues, understanding the underlying causes of environmental issues, knowledge of potential action strategies, and insight into the interconnection between humans and the environment (Jensen, 2002). In this context, it is important to distinguish between different types of environmental knowledge. Objective (factual) knowledge reflects correct information about environmental systems and problems, while subjective knowledge refers to individuals’ perceived familiarity or confidence in their understanding, which may diverge from actual objective knowledge (Shi et al., 2016). Moreover, different types of objective knowledge, such as causal knowledge, knowledge of physical characteristics, or knowledge about the consequences of climate change, may relate differently to psychological outcomes (Shi et al., 2016; Tobler et al., 2012). These distinctions are conceptually important because previous research has shown that subjective knowledge may interact differently with emotional responses and a sense of agency than factual knowledge alone (Geiger et al., 2019).

**FIGURE 1:**
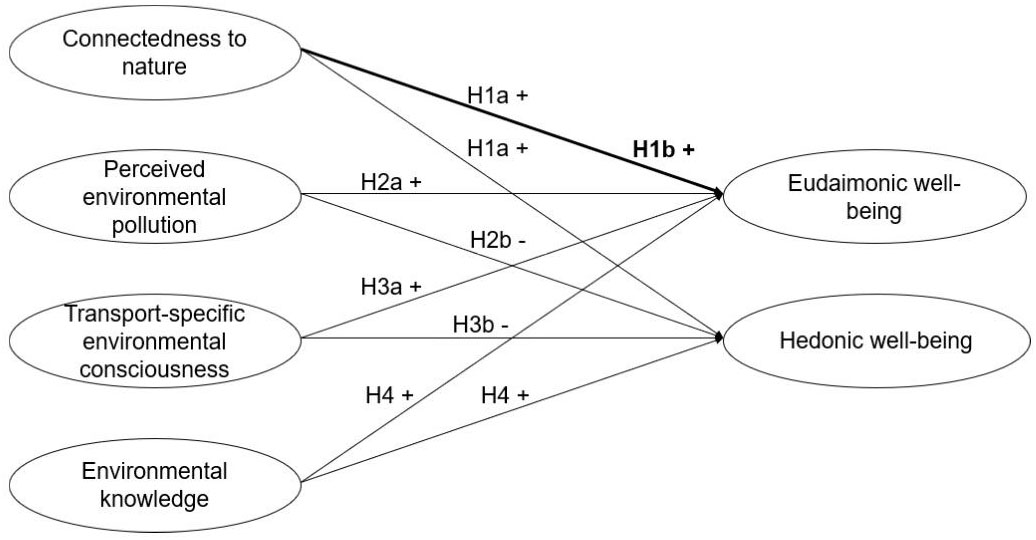
Hypotheses overview. H1b is represented by the bold path, indicating a stronger relationship between connectedness to nature and eudaimonic well-being.

Recent research has begun to examine whether and how environmental knowledge is related to well-being, though studies remain scarce. A growing body of work suggests that environmental knowledge may contribute to well-being primarily through indirect pathways, such as alleviating climate change anxiety (Zacher and Rudolph, 2023). Climate change anxiety, characterized by persistent worry and distress over environmental degradation and its implications, has been shown to negatively affect mental health, contributing to conditions such as depression, generalized anxiety, and psychological distress (McKnight and Kashdan, 2009; Nisbet et al., 2011). Thus, increased factual environmental knowledge may reduce climate change anxiety, as shown by Zacher and Rudolph (2023), and might improve well-being. Similarly, Thomson and Roach (2023) found that factual environmental knowledge plays a role in the relationships between connectedness to nature, climate anxiety, climate action, and mental health outcomes, operationalized through measures of ill-being (e.g., psychological distress, depression). Notably, they reported a negative correlation between environmental knowledge and climate anxiety, supporting the idea that knowledge may represent a buffer against environmental distress (Thomson and Roach, 2023). Additionally, Shi et al. (2016) showed that the type of factual knowledge matters: while causal knowledge about climate change was positively associated with environmental concern, knowledge of physical characteristics was unrelated or even negatively related. Since environmental concern is closely linked to emotional and cognitive responses affecting well-being (Clayton, 2020), these findings suggest that not all knowledge domains have the same psychological implications. Findings show that environmental knowledge may influence well-being through multiple pathways. However, knowledge alone may be insufficient for fostering agency or sustained pro-environmental action, which also requires perceived behavioral control and personal responsibility (Bamberg and Möser, 2007). First, by enhancing individuals’ understanding of environmental challenges and potential solutions, environmental knowledge could foster a sense of agency and control, which are positively linked to well-being. Second, it may help to mitigate climate change anxiety, reducing its negative psychological impact. Taken together, previous research highlights the complexity of how environmental knowledge relates to psychological outcomes. While some findings point to negative or null associations for factual knowledge, others show that environmental knowledge can reduce climate-related anxiety and psychological distress. While prior studies have mostly addressed indirect effects or ill-being, the present study explicitly tests direct associations between environmental knowledge and well-being, offering new insights into its potential psychological benefits. Based on this rationale, we propose the following hypothesis (see Figure 1):

H4: Environmental knowledge is positively associated with eudaimonic and hedonic well-being.

## 3 Material and methods

### 3.1 Data collection

Primary data were collected through an online survey designed for football fans of a professional German third-division football club (Arminia Bielefeld). Participation was restricted to individuals aged 18 or older. Arminia Bielefeld is a professional football club from East Westphalia with a strong regional identity and a deeply rooted tradition. The club and its fan culture are characterized by an explicit commitment to inclusion, anti-discrimination, and social responsibility, actively opposing racism, sexism, and homophobia while promoting accessibility, integration, and civic engagement (Arminia Bielefeld, n.d.-a; Arminia Bielefeld, n.d.-b; Arminia Bielefeld, n.d.-c; Kirschneck and Uhlig, 2005). In addition, the club pursues ambitious environmental goals, including the use of renewable energy in its stadium and reforestation initiatives in the Teutoburg Forest, which reflect a broader ethos of ecological awareness (Arminia Bielefeld, n.d.-a). Today, over 240 officially registered fan clubs, most located within a 100-km radius of Bielefeld, form a socially conscious and passionate supporter base (Arminia Bielefeld, n.d.-c). The online survey was conducted using SoSci Survey^1^, a platform designed for online research in social science, and took place between 25 January and 15 February 2024. As an incentive, participants could voluntarily enter a self-organized lottery upon completing the survey. Three winners were drawn: first place received a football jersey, second place a 3D jigsaw puzzle of the football club’s home stadium, and third place a personalized coffee mug.

The survey link was distributed via various online channels, including social media, club contacts, and emails sent by fan clubs to their members. In sports research, studies have previously employed convenience sampling alongside top-down recruitment strategies for data collection (Thormann and Wicker, 2021a; Thormann and Wicker, 2021b). This approach resulted in 2,554 clicks on the survey link, with 1,562 fans starting the survey and 844 completing it. After data cleaning and plausibility checks (e.g., short completion time and straight-lining, indicating lack of attention), 839 fans were left for the empirical analysis.

### 3.2 Questionnaire and variables

The survey was designed as a standardized online questionnaire. The questionnaire began with an introduction informing participants about the study’s purpose, the voluntary nature of participation, and the confidentiality of their responses. Table 1 provides an overview of all variables used in the empirical analysis. The next paragraphs describe the two well-being measures (eudaimonic and hedonic) as well as several environmental constructs, including their sources and scale properties, an environmental knowledge quiz, and sociodemographic control variables.

**TABLE 1:** Overview of variables and summary statistics (n = 839).

Variable name	Measurement	Mean	SD
Eudaimonic well-being	Mean index of the flourishing scale (items see Table 2; 1 = low well-being; 7 = high well-being)	5.69	0.72
Hedonic well-being	Mean index of the satisfaction with life index (items see Table 2; 1 = low well-being; 7 = high well-being)	5.13	0.99
Connect_to_nat	Mean index of the emotional affinity toward nature scale (items see Table 3; 1 = low connectedness to nature; 6 = very high connectedness to nature)	4.31	0.75
Per_env_poll	Mean index of the perception of the neighborhood as polluted (items see Table 3; 1 = no pollution; 5 = very high pollution)	1.53	0.59
Transport-specific env_consc	Mean index of transport-specific environmental consciousness (items see Table 3; 1 = low environmental consciousness; 5 = very high environmental consciousness)	3.11	0.86
Env_know	Total correct responses in the environmental knowledge quiz (Questions see Table 4; range 0–7)	4.64	1.23
Age	Respondent’s age (in years)	28.11	9.87
Age_sq	Age squared (= Age*Age)	887.46	733.45
Male_gender	Respondent’s self-assessed gender (1 = male; 0 = female)	0.710	
Low_edu	Respondent’s highest educational degree is below A-levels (1 = yes; 0 = no)	0.347	
University entrance qualifications	Highest educational degree is university entrance qualification/A-levels (1 = yes; 0 = no)	0.347	
University	Highest educational degree is university or university of applied sciences degree (1 = yes; 0 = no)	0.306	
Working	Respondent has some form of employment, including full-time, part-time and short-time work, and self-employment (1 = yes; 0 = no)	0.666	
Low income	Personal monthly net income is below 1,000€ (1 = yes; 0 = no)	0.317	
Lower middle income	Personal monthly net income is between 1,001€ and 2,000€ (1 = yes; 0 = no)	0.206	
Upper middle income	Personal monthly net income is between 2,001€ and 3,000€ (1 = yes; 0 = no)	0.319	
High income	Personal monthly net income is between 3,001€ and 4,000€ (1 = yes; 0 = no)	0.098	
Very high income	Personal monthly net income is above 4,000€ (1 = yes; 0 = no)	0.060	
Disability	Respondent has a physical or mental disability (1 = yes; 0 = no)	0.052	

For the measurement of eudaimonic well-being (Table 2), the study used the flourishing scale (Diener et al., 2010). This validated scale was selected because it provides a concise yet comprehensive measure of psychological well-being that aligns with established theoretical frameworks (Ryff and Keyes, 1995; Ryff, 1989). Compared to alternative measures (e.g., psychological well-being scale) (Ryff and Keyes, 1995; Ryff, 1989), the flourishing scale was chosen due to its brevity, strong psychometric properties, and applicability across diverse populations (Diener et al., 1985; Diener et al., 2010; Waterman, 2008). The flourishing scale measures eudaimonic well-being with seven statements on a seven-point Likert scale (1 = strongly disagree; 7 = strongly agree), including statements about relationships with others, purpose and meaning in life, and environmental mastery, reflecting facets of eudaimonic well-being (Aristotle, 1925; Diener et al., 2010; Waterman, 2008). In the present study, the scale showed very good reliability with a Cronbach’s α of 0.843; this interpretation follows commonly accepted thresholds (Hair et al., 2018). A mean index variable for eudaimonic well-being (Eudaimonic well-being) was created by averaging the responses across all seven items. Table 2 provides an overview of all items of the scale and the mean index.

**TABLE 2:** Eudaimonic and hedonic well-being measures.

Items (1 = strongly disagree; 7 = strongly agree)	Mean	SD	Cronbach’s α
Eudaimonic well-being (mean-index)	5.69	0.72	0.843
I am competent and capable in the activities that are important to me	6.01	0.84	
My social relationships are supportive and rewarding	5.99	1.05	
People respect me	5.83	0.89	
I am engaged and interested in my daily activities	5.81	0.97	
I am a good person and live a good life	5.68	1.03	
I actively contribute to the happiness and well-being of others	5.51	1.03	
I lead a purposeful and meaningful life	5.46	1.09	
I am optimistic about my future	5.28	1.35	
Hedonic well-being (mean-index)	5.13	0.99	0.853
I am satisfied with my life	5.51	1.14	
The conditions in my life are excellent	5.41	1.15	
So far I have gotten the important things I want in life	5.17	1.28	
In most ways my life is close to my ideal	4.96	1.13	
If I could live my life over, I would change almost nothing	4.62	1.48	

Fan’s hedonic well-being was captured with the life satisfaction scale from Diener et al. (1985). This widely validated scale was selected over single-item life satisfaction measures to enhance reliability and validity. While single-item measures provide a simple assessment, they cannot capture the more complex nature of life satisfaction and are more prone to measurement error (Lucas and Donnellan, 2012). The satisfaction with life scale measures hedonic well-being with five statements about individuals’ perceptions about their present lives on a seven-point Likert scale (1 = strongly disagree; 7 = strongly agree). This measure was used in previous investigations to capture hedonic well-being (Capaldi et al., 2014; Pritchard et al., 2020). The scale demonstrated very good internal consistency in the present study (Cronbach’s α = 0.853), in line with accepted criteria (Hair et al., 2018). To construct an index variable for hedonic well-being (Hedonic well-being), the mean score across the five items was calculated. The full item battery and the mean index are displayed in Table 2.

Turning to the measurement of the independent variables, the present study relied on validated scales from previous research to assess connectedness to nature, perceived environmental pollution, transport-specific environmental consciousness, and environmental knowledge (Kals et al., 1999; Preisendörfer, 1999). These constructs were selected based on their theoretical relevance for pro-environmental behavior (Kollmuss and Agyeman, 2002) and, in the context of this study, well-being, as well as their contextual fit with sport-related travel behavior (Loewen and Wicker, 2021; Pritchard et al., 2020; Thormann et al., 2022). Together, they capture both emotional and cognitive factors connected to the natural environment in a way that aligns with the study’s objectives. Connectedness to nature was measured using the emotional affinity toward nature scale (Kals et al., 1999), a validated scale assessing respondents’ emotional attachment to nature. This scale was chosen because it specifically captures the emotional aspect of human-nature relationships (Kals et al., 1999; Mayer and Frantz, 2004). Compared to alternative measures such as the connectedness to nature scale (Mayer and Frantz, 2004) or the nature relatedness scale (Pritchard et al., 2020), the emotional affinity scale provides a stronger focus on affective attachment. The scale consists of ten items, each rated on a six-point Likert scale (1 = completely disagree; 6 = completely agree). In the current sample, the scale yielded a Cronbach’s α of 0.836, which is considered very good reliability (Hair et al., 2018). To construct the index variable for connectedness to nature (Connect_to_nat), the mean of the ten items was calculated. Table 3 displays all scale items and the mean index.

**TABLE 3:** Overview of environmental factor measurement.

Items	Mean	SD	Cronbach’s α
**Connect_to_nat (mean-index; 1 = strongly disagree; 6 = strongly agree)**	**4.31**	**0.75**	**0.836**
When I spend time in nature I feel free and easy	5.12	0.84	
When surrounded by nature I get calmer and I feel at home	4.97	0.94	
I have the feeling I can live my life to the full in nature	4.77	0.93	
I do not feel especially at ease whenever I spend time in nature^a^	4.58	1.44	
I feel relaxed and have a pleasant feeling of intimacy when spending time in nature	4.52	1.07	
By getting in touch with nature today I have the feeling of the same origin	4.49	1.16	
Whenever I spend time in nature I do not experience a close connection to it^a^	4.04	1.29	
Sometimes when I feel unhappy I find solace in nature	3.76	1.40	
I am often very much absorbed through nature and I do not notice how time goes by	3.64	1.26	
By direct contact with nature I feel respect for its uniqueness	3.26	1.31	
**Per_env_poll (mean-index; 1 = no pollution; 5 = very high pollution)**	**1.53**	**0.59**	**0.819**
Litter	2.31	1.22	
Street traffic noise	1.72	1.01	
Car exhaust fumes	1.60	0.98	
Poor air quality	1.57	0.96	
Train noise	1.30	0.71	
Emissions/wastewater from factories	1.29	0.78	
Industrial and commercial noise	1.27	0.72	
Airplane noise	1.16	0.54	
**Transport-specific env_consc (mean-index; 1 = strongly disagree; 5 = strongly agree)**	**3.11**	**0.86**	**0.758**
If using a car is unavoidable and it’s feasible, I am always willing to join a carpool	4.00	1.15	
It annoys me that many people who could use buses, trains, or bicycles prefer to drive out of habit	3.26	1.34	
For environmental reasons, I try to travel by car as little as possible, whether as a driver or passenger	2.96	1.28	
In Germany, cars are definitely among the biggest polluters	2.76	1.12	
Environmentalists criticize car drivers too one-sidedly^a^	2.60	1.17	

^a^Item recoded.

To measure perceived environmental pollution, the present study adopted an eight-item scale developed by Preisendörfer (1999). The scale captures respondents’ perceptions of various types of environmental pollution, including air pollution, noise pollution, littering, and smell disturbances. This scale was selected over single-indicator pollution measures because it captures multiple dimensions. Each item was rated on a five-point Likert scale (1 = no pollution; 5 = very high pollution). The scale had high internal consistency in this study (Cronbach’s α = 0.819), consistent with standard benchmarks (Hair et al., 2018). The mean index variable for perceived environmental pollution (Per_env_poll) was computed by averaging all eight items. Table 3 provides an overview of all items and the mean index.

Transport-specific environmental consciousness was measured using a five-item scale adapted from Preisendörfer (1999). The scale assesses three core dimensions of transport-related environmental consciousness – affective, conative, and cognitive. Previous research on fans’ well-being only employed measures of general environmental consciousness (Thormann et al., 2022), not transport-specific measures. The present study used this scale due to the substantial environmental impact of transport in the context of football events. Each item was rated on a five-point Likert scale (1 = completely disagree; 5 = completely agree). The Cronbach’s α for this scale was 0.758, which reflects good internal reliability according to established guidelines (Hair et al., 2018). The index variable for transport-specific environmental consciousness (Transport-specific env_consc) was created by averaging the responses across the five items. Table 3 gives an overview of the items and the index.

Environmental knowledge was assessed using a self-constructed seven-question quiz. Each question had four possible answers in a single-choice design. This approach was chosen because existing scales focus on recycling or general environmental literacy as well as subjective assessments of environmental knowledge (Geiger et al., 2019). However, self-reported knowledge can be prone to biases, such as overestimation or underestimation of actual understanding (Clayton, 2020; Geiger et al., 2019). Also, self-assessments of knowledge often diverge from individuals’ actual objective knowledge (Effeney and Davis, 2013; Jacobs and Roodenburg, 2014), which is also evident in complex domains like sustainability (Effeney and Davis, 2013). To avoid these distortions and ensure an objective assessment, this study directly tested respondents’ knowledge of sustainability and transport-related environmental issues in the German context. The environmental knowledge quiz in the present study measures cause knowledge and knowledge about the physical characteristics of climate change and the environment (Lucas and Donnellan, 2012; Nisbet et al., 2011). As an indicator of respondents’ environmental knowledge, the sum of correct answers to the quiz was used. This resulted in possible scores between zero and seven. Table 4 presents the questions of the environmental knowledge quiz, the correct answers, and the resulting environmental knowledge variable (Env_know).

**TABLE 4:** Env_know quiz.

Questions and answer choices				Correct answers in percent
Which of the following statements is true regarding electric vehicles compared to conventional internal combustion engines? Electric vehicles …				95.4
…*produce no direct* *greenhouse gas emissions*	…create more air pollution	…cause more traffic jams	…contribute more to noise pollution	
What is a common greenhouse gas emission in road traffic?				93.6
Nitrogen (N_2_)	Oxygen (O_2_)	*Carbon dioxide (CO_2_)*	Hydrogen (H_2_)	
Which mode of transport contributes the most to relieving the environment by optimizing traffic flow in cities?				75.1
Electric cars	Cars	SUVs	*Buses*	
What is the primary contribution of the transport sector to the spread of microplastics in the oceans?				54.9
Plastic packaging from car accessories	Ship operation	Plastic from car seats	*Abrasion from tires*	
Which mode of transportation produces the lowest CO_2_ emissions per passenger kilometer?				54.4
Car	*Train (local)*	Bus (local)	Tram	
What percentage of annual total emissions in Germany is accounted for by the transport sector?				48.4
Approximately 1%	Approximately 8%	*Approximately 20%*	Approximately 34%	
Approximately how much CO_2_ emissions could be saved annually in road traffic by a speed limit of 120 km/h on German highways?				42.2
1.3 million tons of CO_2_	*4.5 million tons of CO_2_*	4.2 million tons of CO_2_	20.6 million tons of CO_2_	

Correct answers displayed in italic.

To account for potential confounding factors, the analysis included several sociodemographic control variables following prior research on eudaimonic and hedonic well-being (Aristotle, 1925; Diener et al., 1985; Fredrickson, 2004; Huta and Ryan, 2010; Huta, 2015; Ryff, 1989; Waterman, 2008). These variables were chosen based on their relationships with well-being outcomes, ensuring that the observed effects of factors connected to the natural environment are not biased by systematic differences in personal characteristics. These control variables are: age, gender, education, employment status, income, and disability, which are presented in Table 1.

Age has been recognized as a predictor of both hedonic and eudaimonic well-being, but its relationship is often non-linear. Research suggests that hedonic well-being follows a U-shaped pattern across the lifespan, with lower levels in midlife and higher levels in younger and older adulthood (Effeney and Davis, 2013; Huang and Humphreys, 2012). In contrast, eudaimonic well-being shows a more complex pattern, with autonomy and environmental mastery increasing with age, while purpose in life and personal growth tend to decline (Blanchflower and Oswald, 2008; Diener et al., 2010; Springer et al., 2011). To account for these non-linear associations, age (Age) and the squared term of age (Age_sq) were included in the analysis.

Gender differences in well-being have been extensively studied, with mixed findings. Some studies suggest that women report higher levels of life satisfaction than men at a younger age (Blanchflower and Oswald, 2004; Tay et al., 2014). Others indicate that men report higher life satisfaction than women at an older age (Pinquart and Sörensen, 2001). For eudaimonic well-being, women have been found to score higher on positive relations and personal growth, but studies using composite measures of eudaimonic well-being have found lower overall levels in women compared to men (Marks, 1996). Given these variations, a binary gender variable (Male_gender) was included as a control.

Typically, education is positively associated with well-being (Tan et al., 2020), however, findings are mixed regarding whether eudaimonic or hedonic well-being benefits more from education. Higher educational attainment has been consistently associated with greater life satisfaction, as it enhances access to financial stability and social resources (Boehm et al., 2015). Similarly, education appears to be correlated to all six eudaimonic well-being dimensions, with higher education leading to better well-being (Ryff, 2016). To account for these differences, three levels of education were included in the analysis: low education levels (Low_edu), university entrance qualification (University entrance qualifications), and having completed a university degree (University).

Most research on employment and well-being has focused on job satisfaction rather than employment status itself. While unemployment is generally linked to lower life satisfaction and increased psychological distress (Kinnunen et al., 2006), the present study suggests that being employed may also negatively impact hedonic well-being due to job-related stress and work-life conflict. Given the lack of extensive research on employment status in this context, working was included as a binary control variable (Working) to account for its potential effects on well-being.

Income is a predictor of well-being, particularly in hedonic well-being research (Baumeister et al., 2013; Kahneman and Deaton, 2010). Income tends to have a stronger impact on hedonic than eudaimonic well-being, supporting findings that economic dependence influences life satisfaction more strongly (Baumeister et al., 2013; Kahneman and Deaton, 2010). To capture potential threshold effects, net income was classified into five groups: Low income, Lower middle income, Upper middle income, High income, and Very high income (Table 1).

Disability can significantly impact both hedonic and eudaimonic well-being, affecting life satisfaction, autonomy, and environmental mastery (Mishra et al., 2019). Prior research suggests that individuals with disabilities often experience lower hedonic well-being, particularly due to barriers to social participation and employment (Mishra et al., 2019). To account for these differences, a binary variable for disability (Disability) was included.

### 3.3 Empirical analysis

The empirical analysis consisted of three steps. First, descriptive statistics were calculated to provide an overview of the sample structure. Second, seemingly unrelated regression (SUR) models were employed, as the dependent variables – eudaimonic and hedonic well-being – are conceptually related (Huta, 2015; Ryan and Deci, 2001) and showed a moderate and statistically significant correlation (ρ = 0.64; *p* < 0.001). This correlation suggests the presence of correlated error terms, which violate the assumption of error independence in separate regression models (Hair et al., 2018). The Breusch-Pagan test confirmed a significant association of error terms (*p* < 0.001), justifying the use of a SUR model. The SUR model included connectedness to nature, perceived environmental pollution, transport-specific environmental consciousness, and environmental knowledge as the independent variables of interest, while the remaining variables from Table 1 were included as controls.

To assess potential multicollinearity of the independent variables, correlation coefficients and variance inflation factors were examined. All correlation coefficients were below 0.8. This also applied to the independent variables of interest: Despite the outlined conceptual linkages between the key independent variables, correlation analyzes revealed only small to moderate associations between them (*r* = 0.02–0.30; see Supplementary Table 1), suggesting they can be treated as statistically independent predictors. VIF values remained under the critical threshold of 10 as suggested by Ryff and Keyes (1995), ranging from 1.04 to 2.81 for the independent variables of interest. This supports the inclusion of all four environmental variables in the model, as they provide unique contributions despite conceptual overlap. By construction, higher VIFs were only observed for age (VIF: 33.02) and age squared (VIF: 29.46), which were included to account for non-linear effects.

Third, a series of Wald tests were conducted in StataCorp (2023) following the SUR model estimation to evaluate the hypotheses summarized in Figure 1. These tests assessed whether the coefficients of the four key predictors, connectedness to nature, perceived environmental pollution, transport-specific environmental consciousness, and environmental knowledge, differed significantly between the two outcome variables (eudaimonic and hedonic well-being). In addition to the main predictors, coefficient differences for control variables (e.g., age, gender, employment status) were also tested. The tests were implemented using standard test (e.g., “test [hedonic well-being]Predictor = [eudaimonic well-being]Predictor”) commands after the ‘sureg’ estimation command in Stata.

## 4 Results

Table 1 summarizes the sample structure. For the dependent variables, the respondents’ eudaimonic well-being was on average 5.69 (on a scale from 1 to 7) and therefore slightly higher than the hedonic well-being with a mean value of 5.13.

The descriptive statistics of the independent variables provide the following insights into how factors connected to the natural environment are represented in the sample. With an average of 4.31 on a scale from 1 to 6, respondents tended to agree with the connectedness to nature statements, suggesting a moderate level of emotional attachment to nature. Respondents perceived their living environment as little polluted as indicated by an average of 1.53 on a scale from 1 (no pollution) to 5 (very high pollution), which implies that environmental pollution was not perceived as a major issue in their surroundings. The average transport-specific environmental consciousness score was 3.11 (on a scale from 1 to 5). Thus, on average, respondents neither agreed nor disagreed with the transport-specific environmental consciousness statements. The environmental knowledge of respondents was fairly high, as they were able to answer an average of 4.64 out of 7 questions correctly in the environmental knowledge quiz.

For the demographic control variables, the average age of respondents was 28.11 years, and the sample consisted of mostly male respondents (71%). In terms of education, 34.7% of respondents had a university entrance qualification as their highest degree, 34.7% had lower education degrees, and 30.6% had graduated from a university or university of applied sciences. The majority (66.6%) of respondents worked in some form of employment. Regarding net income, the largest portion of the sample (31.9%) fell into the upper middle income group, with an average monthly net income between €2,001 and €3,000. This was followed by the low income group (monthly net income below €1,000), which comprised 31.7% of respondents. The lower middle income group accounted for 20.6% of respondents, with monthly net incomes between €1,001 and €2,000, followed by 9.8% in the high income group (€3,001–€4,000) and 6.0% in the very high income group (over €4,000). 5.2% of respondents reported having a physical or mental disability.

**FIGURE 2:**
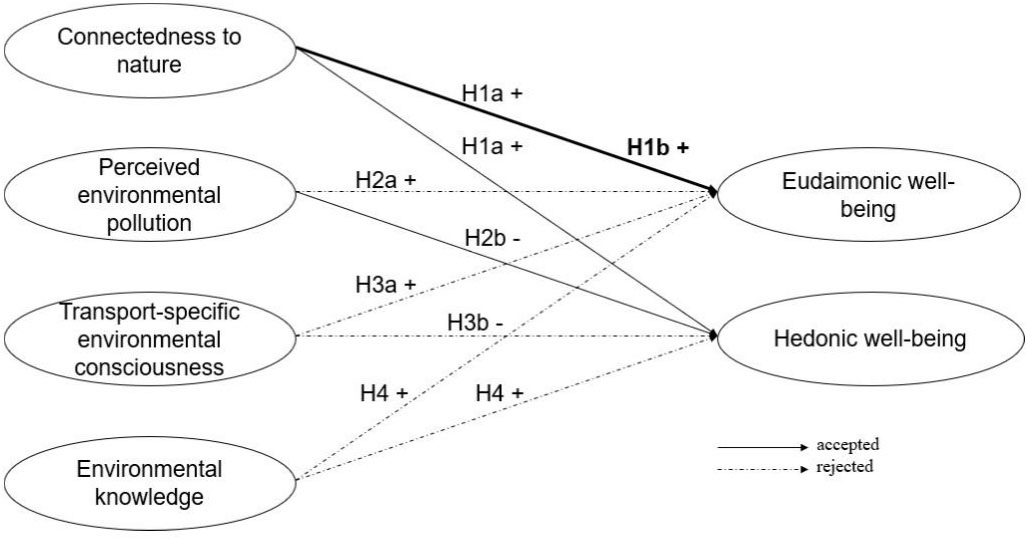
Hypotheses testing. H1b is represented by the bold path, indicating a stronger relationship between connectedness to nature and eudaimonic well-being.

**FIGURE 3:**
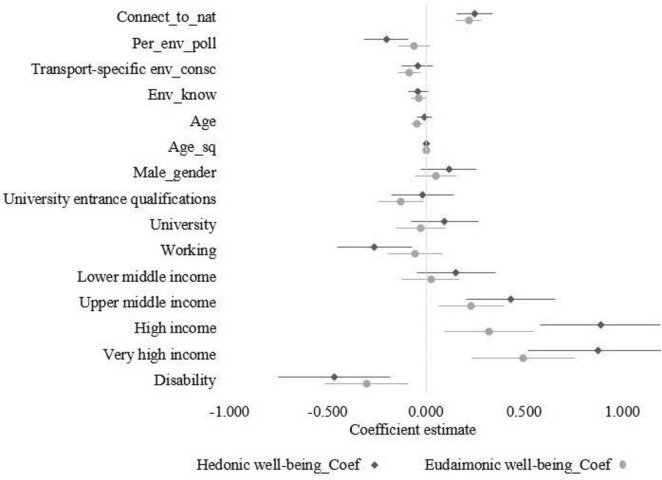
Forest plot regression coefficients.

To address the RQ, Table 5 presents the results of the SUR model and Wald test. Figure 2 provides an overview of the hypotheses and indicates whether each was accepted or rejected. Additionally, Figure 3 outlines the regression coefficients for the two models. Connectedness to nature was positively and significantly associated with both eudaimonic (β = 0.217; *p* < 0.001) and hedonic well-being (β = 0.249; *p* < 0.001). A one-unit increase in connectedness to nature corresponded to a 0.217-unit increase in eudaimonic well-being and a 0.249-unit increase in hedonic well-being. This supports H1a and the assumption that higher connectedness to nature leads to eudaimonic and hedonic well-being. While the association was slightly stronger with hedonic well-being, the Wald test (χ*^2^* = 0.79; *p* = 0.373) indicated no statistically significant difference between the two coefficients, leading to the rejection of hypothesis H1b. These findings suggested that stronger connectedness to nature was linked to both greater purpose-driven well-being and immediate happiness.

**TABLE 5:** Seemingly unrelated regression models for eudaimonic and hedonic well-being (*n* = 839).

	Eudaimonic well-being	Hedonic well-being	Wald test (χ ^2^)
Connect_to_nat	0.217^***^	0.249^***^	0.79
Per_env_poll	−0.061	−0.203^***^	10.72^***^
Transport-specific env_consc	−0.085^**^	−0.043	1.83
Env_know	−0.036	−0.039	0.03
Age	−0.046^**^	−0.007	7.57^**^
Age_sq	0.000*	0.000	6.00*
Male_gender	0.050	0.117	1.51
Low_edu	Ref.	Ref.	-
University entrance qualifications	−0.128*	−0.016	3.34
University	−0.025	0.096	3.36
Working	−0.057	−0.261^**^	7.79^**^
Low income	Ref.	Ref.	-
Lower middle income	0.024	0.153	2.75
Upper middle income	0.232^**^	0.432^***^	5.31*
High income	0.322^**^	0.890^**^	22.96^**^
Very high income	0.497^**^	0.876^**^	7.61^**^
Disability	−0.302^**^	−0.465^**^	2.18
Constant	6.123	4.681	-
Pseudo *R*^2^	0.10	0.12	
χ^2^	89.44^**^	115.83^**^	
Breusch-Pagan test χ^2^	364.23^**^		
r	0.66		

*p < 0.05;

^**^p < 0.01;

^**^p < 0.001; displayed are the unstandardized coefficients; Ref. = reference category.

Perceived environmental pollution showed no significant relationship with eudaimonic well-being (β = −0.061; *p* = 0.144) but was significantly negatively associated with hedonic well-being (β = −0.203; *p* < 0.001). This means that a one-unit increase in perceived environmental pollution corresponded to a 0.203-unit decrease in hedonic well-being. Consequently, H2a for eudaimonic well-being was rejected, while H2b for the hedonic well-being dimension was accepted. The Wald test (χ*^2^* = 10.72; *p* < 0.001) confirmed a statistically significant difference in the estimated coefficients, indicating that perceived environmental pollution had a stronger negative association with hedonic well-being compared to eudaimonic well-being.

In contrast to H3a, transport-specific environmental consciousness was not positively but significantly negatively associated with eudaimonic well-being (β = −0.085; *p* = 0.005). A one-unit increase in transport-specific environmental consciousness corresponded to a 0.085-unit decrease in eudaimonic well-being, suggesting that individuals with greater awareness of transport-related environmental concerns experience lower levels of purpose-driven well-being. However, transport-specific environmental consciousness had no significant negative effect on hedonic well-being (β = −0.043; *p* = 0.299), which leads to a rejection of H3b. Finally, environmental knowledge showed no significant association with well-being, neither with eudaimonic well-being (β = −0.036; *p* = 0.068) nor hedonic well-being (β = −0.039; *p* = 0.142) – and H4 was rejected.

Turning to the sociodemographic controls, only the income group variables and having a disability showed significant associations with both well-being outcomes. Respondents in the upper middle income group and above, with a monthly net income higher than 2,000€, had significantly higher well-being compared to those in the low income group (monthly net income less than 1,000€), which served as the reference category. These associations were significantly stronger for hedonic well-being compared to eudaimonic well-being, as indicated by the Wald test: upper middle income (*p* = 0.021), high income (*p* < 0.001), and very high income (*p* = 0.006). Respondents with a disability reported lower well-being levels for both measures. In addition to the factors affecting both well-being dimensions, certain variables were significantly associated only with one dimension. Specifically, having a university entrance qualification (compared to the reference group of low education) showed a negative association. Furthermore, age exhibited a U-shaped relationship with eudaimonic well-being, as reflected in the negative coefficient for age and the positive coefficient for age squared. The turning point is at the age of 54.0 years. No such effects were evident for the link between age and hedonic well-being. Lastly, working showed no significant association with eudaimonic well-being (β = −0.057; *p* = 0.429) but was significantly negatively associated with hedonic well-being (β = −0.261; *p* = 0.007). The Wald test (χ*^2^* = 7.79; *p* = 0.005) confirmed a statistically significant difference in the estimated coefficients, showing that working in some form of employment had a significantly larger negative relation with hedonic well-being compared to eudaimonic well-being.

## 5 Discussion

The present research aimed to investigate the well-being of football fans, focusing on both eudaimonic and hedonic well-being. It examined the RQ, how factors connected to the natural environment relate to the well-being of football fans.

The findings show that football fans with higher connectedness to nature report significantly higher levels of both eudaimonic and hedonic well-being. This supports prior evidence that nature connectedness is a reliable predictor of well-being (Capaldi et al., 2014; Pritchard et al., 2020), and extends this relationship into the underexplored context of sport fandom. The present study did not find a statistically significant difference between the associations of connectedness to nature with eudaimonic versus hedonic well-being, contrasting earlier findings (Capaldi et al., 2014; Howell et al., 2011; Pritchard et al., 2020). One possible explanation is that previous research did not explicitly differentiate between these two well-being dimensions (Capaldi et al., 2014; Pritchard et al., 2020). Consequently, the previously reported differences may reflect methodological imprecision or a lack of theoretical distinction rather than true differences, which were directly addressed in the current study. The relationship found in this study can be explained with the Biophilia Hypothesis (Wilson, 1984), which suggests that humans have an innate tendency to seek connections with nature, leading to greater psychological fulfillment as it aligns with fundamental evolutionary needs (Mayer and Frantz, 2004). Likewise, stress reduction theory (Ulrich et al., 1991) highlights the immediate physiological and emotional benefits of nature exposure, such as lower cortisol levels and increased positive affect, which could explain why hedonic well-being benefits as much as eudaimonic well-being from connectedness to nature. Although prior research suggested a stronger relationship between connectedness to nature and eudaimonic well-being due to its associations with meaning, purpose, and long-term behavioral patterns, a meta-analysis by Pritchard et al. (2020) found no significant differences in effect sizes between eudaimonic and hedonic well-being, challenging this assumption. One possible explanation for these inconsistencies is that previous studies may have relied on broad indices rather than subdimensions of well-being. For instance, Pritchard et al. (2020) found differences only at the subcomponent level of eudaimonic well-being. This suggests that aggregate indices may obscure nuanced distinctions. While our study confirms the positive association between connectedness to nature and well-being, it challenges the assumption that eudaimonic well-being is more strongly associated with it than hedonic well-being, emphasizing the need for further research to refine our understanding of this relationship.

For perceived environmental pollution, the present findings reached statistical significance for hedonic well-being, with the Wald test indicating a stronger negative effect on hedonic than eudaimonic well-being. This is consistent with prior research suggesting that perceived environmental pollution can function as a psychological stressor, where individuals interpret polluted environments (e.g., air pollution) as a threat to their health (Campbell, 1983). Even in the absence of objectively harmful pollution levels, the awareness or belief in pollution can elicit negative emotions such as sadness, anger, or hopelessness (Li et al., 2018), which undermine hedonic well-being. This aligns with studies that link perceived air quality, noise, and other forms of pollution to reduced life satisfaction and increased negative affect (Bentley et al., 2023; Finell et al., 2024; Hammersen et al., 2016; Hassan and Khalil, 2024; Herrera and Cabrera-Barona, 2020; Li et al., 2018; Rehdanz and Maddison, 2008). The Wald test underscores the differential impact of perceived environmental pollution on the two well-being dimensions. While hedonic well-being is significantly negatively affected, no such relationship was found for eudaimonic well-being, contradicting prior research by Gu et al. (2015). Their study found a positive relationship between air pollution and eudaimonic well-being and posited that pollution could act as a “meaning violation” (56, p. 73), prompting individuals to reaffirm purpose and meaning in other life domains (Heine et al., 2006). This process, known as fluid compensation, suggests that individuals may respond to environmental stressors by reinforcing thoughts of purpose and engaging in meaningful activities, which enhance eudaimonic well-being. The discrepancy between our findings and Gu et al. (2015) can be attributed to differences in the measurement of the pollution and well-being variables. For the measurement of pollution, they focused solely on air pollution, and their assessment of eudaimonic well-being relied on the Meaning in Life Questionnaire, which captures only a single dimension of this construct (Gu et al., 2015). In contrast, our study utilized a composite measure of perceived environmental pollution – including air, noise, smell, and litter – and a multi-item multidimensional measure of eudaimonic well-being. This broader approach may dilute or obscure relationships that are more context-specific, such as those observed for air pollution alone. Additionally, the present study’s finding of no significant relationship between perceived environmental pollution and eudaimonic well-being could reflect the complexity of how individuals navigate and interpret environmental stressors. While pollution might inspire activism or purpose-driven behavior for some individuals, others may lack the resources or opportunities to engage in such compensatory actions, thus limiting potential gains in eudaimonic well-being.

Transport-specific environmental consciousness exhibited a significant negative relationship with eudaimonic well-being but was not associated with hedonic well-being. The Wald test did not confirm a stronger impact on eudaimonic well-being, contrasting prior research that environmental consciousness can influence well-being positively or negatively, depending on cognitive and emotional framing (Ferrer-I-Carbonell and Gowdy, 2007). The negative association with eudaimonic well-being suggests that individuals aware of the environmental impact of their travel choices experience cognitive dissonance, as frequent travel to sporting events may conflict with their values, reducing purpose and fulfillment. Additionally, internalized distress such as eco-anxiety and guilt (Schwartz et al., 2022; Searle and Gow, 2010) may further undermine well-being. A lack of perceived agency could also play a role. If individuals feel that choosing sustainable transport is insufficient to address climate change, they may develop a sense of powerlessness, negatively affecting their environmental mastery (Huta and Ryan, 2010). These findings align with Thormann et al. (2022), who attribute the negative impact of environmental consciousness on well-being to two factors. First, high scores in the affective dimension, which includes worry and distress about environmental issues, could contribute to lower well-being (Ferrer-I-Carbonell and Gowdy, 2007; Rehdanz and Maddison, 2008). Second, broader climate concerns, such as the International Panel on Climate Change (IPCC) projection that the 1.5°C goal will not be met, may exacerbate feelings of helplessness, further decreasing well-being (IPCC, 2018; Thormann et al., 2022). The lack of an effect on hedonic well-being suggests that the enjoyment of attending football matches outweighs concerns about sustainability (Binder and Blankenberg, 2016). Prior research shows that the impact of environmental consciousness on well-being depends on connotations: Those who view environmental action positively report higher well-being, while those who associate it with threats and destruction report lower well-being (Ferrer-I-Carbonell and Gowdy, 2007; Nisbet et al., 2011). Moreover, environmental consciousness may carry psychological costs. Especially for younger fans, heightened consciousness of ecological problems can trigger emotional distress or eco-anxiety (Clayton, 2020), which may partly explain the observed negative associations with well-being. This highlights the dual role of environmental consciousness as both a motivator for change and a source of psychological strain.

Environmental knowledge demonstrated no correlation with eudaimonic and hedonic well-being among football fans. This result is in contrast to prior research and the theoretical assumptions, which have suggested that environmental knowledge may positively impact well-being by reducing climate change anxiety (Zacher and Rudolph, 2023). Specifically, environmental knowledge can alleviate feelings of anxiety, depression, and distress stemming from concerns about climate change (Schwartz et al., 2022; Searle and Gow, 2010). Without opportunities to act upon this knowledge, individuals may feel helpless or overwhelmed, which could counteract any potential benefits (Thomson and Roach, 2023). Another potential factor is that environmental knowledge may primarily influence climate anxiety rather than directly enhancing well-being (Thomson and Roach, 2023). This suggests that its effects might be more pronounced in negative psychological constructs – such as distress, worry, or eco-anxiety – rather than in positive well-being measures like life satisfaction or personal growth. Moreover, the Wald test results did not confirm a differential impact of environmental knowledge on eudaimonic versus hedonic well-being, suggesting that its potential effects are not stronger for one dimension of well-being over the other. These null findings may stem from limitations in how environmental knowledge was measured. Although the seven-item quiz allowed for an objective assessment of factual knowledge, it did not capture the multidimensional nature of environmental knowledge (Tobler et al., 2012). Previous research has shown that subjective, self-assessed knowledge may relate differently to psychological outcomes, particularly when individuals overestimate their own understanding (Geiger et al., 2019; Shi et al., 2016). Moreover, the specific type of knowledge assessed appears to matter: while causal knowledge about climate change tends to increase concern about climate change, knowledge of physical characteristics can have no or even negative associations with concern (Shi et al., 2016). Conceptual frameworks further emphasize the complexity of environmental knowledge. Tobler et al. (2012), for instance, differentiate between knowledge of physical processes, causal mechanisms, and expected consequences of climate change. Similarly, Jensen (2002) identifies four dimensions: understanding environmental impacts, identifying causes, knowing action strategies, and grasping the relationship between humans and nature. Against this backdrop, a narrowly focused quiz may fail to account for the full breadth of environmentally relevant knowledge and its psychological implications. The omission of motivational components may further limit explanatory power, helping to account for the absence of significant associations in the present study.

Although the environmental variables were analyzed as independent predictors, their small to moderate correlations and shared conceptual underpinnings suggest they may function as a broader environmental orientation. Future research should explore these dynamics through integrated models, examining potential interaction or mediation effects (e.g., transport-specific environmental consciousness mediating the link between perceived environmental pollution and well-being).

Overall, the models explained 10 and 12% of the variance in eudaimonic and hedonic well-being, respectively. While modest, such levels are common in psychological research, where explained variance typically centers around 40%, reflecting the complexity and contextual dependency of human behavior (Smedslund et al., 2022). Despite the low variance explained, significant associations, especially for connectedness to nature and income, underscore the relevance of distinct factors connected to the natural environment. Future studies could include broader psychological or contextual variables to improve explanatory strength.

While the current sample provides valuable insights into the factors connected to the natural environment among football fans, its demographic skew, primarily young, male, and based in Germany, limits the broader generalizability of the findings. The surveyed fans were affiliated with Arminia Bielefeld, a German third-division club known for its progressive, socially engaged supporter base and regional identity. With mostly local fan clubs this context reflects a specific cultural and environmental ethos that may differ from other clubs, sports, or countries. Future studies should further investigate whether similar patterns exist among fans of other sports, in other national contexts, or within more demographically and ideologically diverse supporter groups.

The findings of this study have implications for football clubs, sponsors, and policymakers aiming to promote environmental sustainability in ways that align with fan culture and well-being. Although the findings are specific to football fans, they may offer valuable insights for other sports contexts as well, particularly where fan identity, collective rituals, and environmental engagement intersect. Contrary to stereotypes of football fans as environmentally indifferent or disruptive (Paché, 2020; Pearson, 2012), the results suggest that fans exhibit moderate environmental awareness – particularly in their connectedness to nature and environmental knowledge – while their transport-specific environmental consciousness and perceived environmental pollution are more neutral. Importantly, the results do not suggest that fans must be catered to improve their well-being, but rather that understanding how fans think and feel about environmental issues can inform more effective sustainability strategies. If football events are to become more environmentally responsible, clubs, sponsors, and policymakers must understand how fans experience environmental concerns and identify levers for action that resonate with their identities and matchday routines. This knowledge is critical for designing measures that reduce pollution and improve sustainability, especially on match days, without alienating fans and while promoting their meaningful engagement. Rather than imposing sustainability measures on fans, these insights offer an opportunity to align environmental initiatives with fan identity and values (Casper et al., 2020; Inoue and Kent, 2012).

Fans show moderate environmental awareness in their connectedness to nature. This suggests that while the foundation for sustainability engagement exists, targeted, fan-centered interventions are needed. For example, the strong link between connectedness to nature and well-being highlights that nature-related elements can serve as emotional entry points for sustainable fan engagement. Clubs could integrate natural elements into stadium architecture, create outdoor fan zones, or organize partnerships with local conservation groups. Moreover, nature-themed matchdays or collaborative initiatives such as tree planting or stadium greening projects could enhance fans’ sense of purpose and identity while contributing to environmental goals.

In terms of perceived environmental pollution, the negative association with hedonic well-being underscores the importance of improving the immediate matchday environment. Measures such as better air quality, noise reduction, and cleaner public spaces can make attending games more enjoyable while also benefiting residents. To ensure the success of such measures, fan inclusion is key: when fans are actively involved in developing and implementing solutions – such as waste reduction campaigns or clean-up actions – they are more likely to support and sustain them (McCullough and Cunningham, 2011). These co-created initiatives can also help reduce tensions between fans and local communities and foster a more cooperative and responsible environmental culture around football events.

The study also show that transport-specific environmental consciousness negatively relates to eudaimonic well-being, pointing to a conflict between fans’ values and behavior (Langseth and Vyff, 2021). Instead of avoiding this tension, clubs and transport providers can offer meaningful alternatives that empower fans: fan-organized carpooling, subsidized public transport, shuttle services, or collaborations with green mobility providers can help reduce matchday emissions and support environmentally friendly travel. These options must be clearly communicated as fan-aligned and identity-affirming, rather than externally imposed restrictions.

Although environmental knowledge was relatively high among fans, this did not translate into higher well-being – indicating that knowledge alone is not enough. Therefore, sustainability campaigns should go beyond information dissemination. Instead, they should include behaviorally informed strategies that encourage active and identity-relevant participation. For example, highlighting and rewarding sustainable fan behavior – through public recognition, competitions, or matchday incentives – may strengthen both motivation and well-being, and could help bridge the gap between awareness and meaningful contribution (Bühren and Daskalakis, 2015; Cayolla et al., 2024).

In sum, the study suggest that football fans are not inherently resistant to environmental action. However, for sustainability measures to succeed, clubs, sponsors, and policymakers must acknowledge the realities of fan culture, reduce barriers to action, and build on existing attitudes and values. Rather than imposing top-down rules, they should co-create spaces where environmental responsibility becomes a visible, valued, and identity-relevant part of football culture – particularly in the highly emotional and behaviorally impactful context of matchdays. Fan participation should be seen not only as a means of implementation but as a central strategy to increase commitment, identification, and long-term impact. This approach promises not only ecological and environmental benefits but also improved relationships between fans, clubs, and host communities.

## 6 Conclusion

This study examined eudaimonic and hedonic well-being of football fans of a football club in the German third-division and the correlations with various factors connected to the natural environment. Key findings indicate that connectedness to nature positively influences both eudaimonic and hedonic well-being, underscoring its role in fostering purpose-driven meaning in life and immediate happiness. In contrast, transport-specific environmental consciousness was negatively associated with eudaimonic well-being, highlighting potential cognitive and emotional burdens tied to awareness of environmental issues and behavioral incongruence. Perceived environmental pollution exhibited no association with eudaimonic well-being but a significant negative association with hedonic well-being, aligning with its role as a psychological stressor.

The present study adds to the existing body of knowledge in well-being research by providing a nuanced understanding of the interplay between eudaimonic versus hedonic well-being and factors connected to the natural environment among football fans. It is among the first in sports research to examine these distinct yet related dimensions of well-being in connection with environmental perceptions and dispositions. Moreover, the present research builds on previous sports research by offering a more structured operationalization and measurement of well-being, incorporating both multi-dimensional eudaimonic and hedonic well-being within a clear theoretical framework. In addition, this work advances theoretical perspectives on well-being by integrating constructs related to the natural environment into established conceptual frameworks. The findings support perspectives such as the Biophilia Hypothesis (suggesting that nature connectedness enhances well-being) and stress reduction theory (explaining the negative impact of perceived pollution on hedonic well-being).

Beyond its contribution to well-being research, the current investigation enhances the understanding of environmental perceptions in sports contexts. By analyzing connectedness to nature, perceived environmental pollution, transport-specific environmental consciousness, and environmental knowledge, it expands knowledge on how football fans engage with sustainability-related issues beyond matchday behaviors. These findings challenge the perception of football fans as environmentally indifferent and highlight the psychological implications of sustainability awareness in sports spectatorship.

This study is not without limitations, which can guide future research. First, the research is based on data from a single football club in Germany, which may limit the extent to which the findings apply to other settings. Future studies should examine different leagues, sports, and cultural contexts to determine whether the observed relationships hold across diverse fan bases and sporting environments. Second, the cross-sectional design limits causal inference. Longitudinal and mixed-method follow up studies could provide insights into how well-being evolves over time, particularly in response to changes in environmentally oriented attitudes and perceptions, either driven by sustainable initiatives from clubs or by broader societal shifts. Third, the study relies on self-reported measures, which may introduce biases such as social desirability. Future research could incorporate physiological or behavioral indicators of well-being, such as heart rate variability or passive smartphone-based mood tracking, to complement self-reports (Reichert et al., 2021). Moreover, scholars should explore potential mediators (e.g., climate anxiety, emotional conflict) and moderators (e.g., fan identity, value alignment) not only between factors connected to the natural environment and well-being, but also among the key independent variables themselves. This could clarify how constructs like connectedness to nature, perceived pollution, or environmental knowledge interact and shape well-being in different fan contexts. Also, social norms (Nyborg et al., 2016), personal values (Casper et al., 2020), and team loyalty (Inoue and Kent, 2012) affect pro-environmental attitudes and behavior, which may also shape how factors connected to the natural environment affect well-being.

## Data Availability

The raw data supporting the conclusions of this article will be made available by the authors, without undue reservation.

## Footnotes

1
https://www.soscisurvey.de/
