# Supplementary material for: How factors connected to the natural environment shape football fans’ eudaimonic and hedonic well-being
Source: Front Psychol. 2025 Sep 30;16:1604029. doi: 10.3389/fpsyg.2025.1604029 (PMC12520019; doi:10.3389/fpsyg.2025.1604029)
Supplement: Supplementary file 1 [file Table_1.docx]

|  | Connect_to_nat | Per_env_poll | Transport-specific env_consc | Env_know |
| --- | --- | --- | --- | --- |
| Connect_to_nat | 1.00 |  |  |  |
| Per_env_poll | 0.166** | 1.00 |  |  |
| Transport-specific env_consc | 0.296** | 0.254** | 1.00 |  |
| Env_know | 0.098** | 0.021 | 0.070* | 1.00 |

Supplementary Material Manuscript 1604029

S1. Correlation coefficients for key independent variables.

Note: **p* < 0.05; ***p* < 0.01; ****p* < 0.001.

S2. Overview of VIF values.

|  | VIF values |
| --- | --- |
| Connect_to_nat | 1.17 |
| Per_env_poll | 1.11 |
| Transport-specific env_consc | 1.23 |
| Env_know | 1.05 |
| Age | 33.02 |
| Age_sq | 29.46 |
| Male_gender | 1.07 |
| University entrance qualifications | 1.45 |
| University | 1.59 |
| Working | 2.06 |
| Lower middle income | 1.70 |
| Upper middle income | 2.81 |
| High income | 2.13 |
| Very high income | 1.81 |
| Disability | 1.04 |

S3. Seemingly unrelated regression models for eudaimonic and hedonic well-being with team loyalty and interaction effects (n=839).

|  | | Eudaimonic well-being | Hedonic well-being |  |
| --- | --- | --- | --- | --- |
| Connect_to_nat | | 0.025 | 0.184 |  |
| Per_env_poll | | -0.442 | -0.808 |  |
| Transport-specific env_consc | | 0.035 | 0.097 |  |
| Env_know | | 0.131 | -0.14 |  |
| Team Loyalty | | -0.147 | -0.214 |  |
| Connect_to_nat × Team Loyalty | | 0.041 | 0.015 |  |
| Per_env_poll Team × Loyalty | | 0.081 | 0.129 |  |
| Transport-specific env_consc × Team Loyalty | | -0.014 | -0.029 |  |
| Env_know × Team Loyalty | | -0.045 | 0.02 |  |
| Age | | -0.047 | -0.008 |  |
| Age_sq | | 0.000^**^ | 0.000 |  |
| Male_gender | | 0.047^*^ | 0.113 |  |
| Low_edu | | Ref. | Ref. |  |
| University entrance qualifications | | -0.123 | -0.017 |  |
| University | | -0.023 | 0.101 |  |
| Working | | -0.049 | -0.256^**^ |  |
| Low income | | Ref. | Ref. |  |
| Lower middle income | | 0.019 | 0.142 |  |
| Upper middle income | | 0.229^**^ | 0.422^***^ |  |
| High income | | 0.32^**^ | 0.875^***^ |  |
| Very high income | | 0.501^***^ | 0.869^***^ |  |
| Disability | | -0.297^**^ | -0.443^**^ |  |
| Constant | | 6.819 | 5.681 |  |
| Pseudo R^2^ | | 0.10 | 0.13 |  |
| χ^2^ | | 96.68^***^ | 120.58^***^ |  |
| Breusch-Pagan test | χ^2^ | 364.00^***^ | | |
|  | r | 0.66 | | |

Note: **p* < 0.05; ***p* < 0.01; ****p* < 0.001; displayed are the unstandardized coefficients; Ref. = reference category.
